# Precision Oncology via NMR-Based Metabolomics: A Review on Breast Cancer

**DOI:** 10.3390/ijms22094687

**Published:** 2021-04-28

**Authors:** Alessia Vignoli, Emanuela Risi, Amelia McCartney, Ilenia Migliaccio, Erica Moretti, Luca Malorni, Claudio Luchinat, Laura Biganzoli, Leonardo Tenori

**Affiliations:** 1 Magnetic Resonance Center (CERM), University of Florence, 50019 Sesto Fiorentino, Italy; vignoli@cerm.unifi.it (A.V.); tenori@cerm.unifi.it (L.T.); 2 Department of Chemistry “Ugo Schiff”, University of Florence, 50019 Sesto Fiorentino, Italy; 3 Department of Medical Oncology, New Hospital of Prato S. Stefano, 59100 Prato, Italy; emanuela.risi@uslcentro.toscana.it (E.R.); amelia.mccartney@uslcentro.toscana.it (A.M.); ilenia.migliaccio@uslcentro.toscana.it (I.M.); erica.moretti@uslcentro.toscana.it (E.M.); luca.malorni@uslcentro.toscana.it (L.M.); laura.biganzoli@uslcentro.toscana.it (L.B.); 4 School of Clinical Sciences, Monash University, Melbourne 3800, Australia; 5 Consorzio Interuniversitario Risonanze Magnetiche di Metallo Proteine (C.I.R.M.M.P.), 50019 Sesto Fiorentino, Italy

**Keywords:** metabolomics, NMR, breast cancer, precision medicine, chemotherapy

## Abstract

Precision oncology is an emerging approach in cancer care. It aims at selecting the optimal therapy for the right patient by considering each patient’s unique disease and individual health status. In the last years, it has become evident that breast cancer is an extremely heterogeneous disease, and therefore, patients need to be appropriately stratified to maximize survival and quality of life. Gene-expression tools have already positively assisted clinical decision making by estimating the risk of recurrence and the potential benefit from adjuvant chemotherapy. However, these approaches need refinement to further reduce the proportion of patients potentially exposed to unnecessary chemotherapy. Nuclear magnetic resonance (NMR) metabolomics has demonstrated to be an optimal approach for cancer research and has provided significant results in BC, in particular for prognostic and stratification purposes. In this review, we give an update on the status of NMR-based metabolomic studies for the biochemical characterization and stratification of breast cancer patients using different biospecimens (breast tissue, blood serum/plasma, and urine).

## 1. Breast Cancer: Why Precision Oncology?

Precision medicine, also called personalized medicine, is an emerging approach for disease treatment and prevention that takes into account genetics, epigenetics, metabolism, environment, and lifestyle of each individual person with the goal to select the optimal therapy for the right patient. In oncology, tumor molecular profiling leads to the identification of patient specific alterations that could inform about the optimal treatments and maximize patient’s survival.

**Figure 1:**
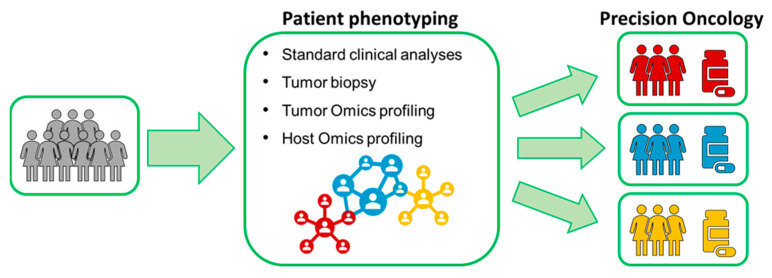
Precision oncology in a nutshell.

For several years breast cancer (BC) has been seen as a single clinical entity and treated with one general approach. However, now it has become extremely clear that BC has to be considered a highly heterogeneous disease with different subclasses. The discovery of endocrine receptors, and the understanding that endocrine therapy significantly improves outcomes in patients with hormone receptor-positive disease, marks the beginning of the target therapy for patients with BC [1,2,3]. By the late 1990s, it was discovered that a subgroup of breast tumors (15–20%) overexpresses the HER2 receptor or have HER2 gene amplification. HER2-positive disease had a dismal outcome until the development of targeted agents, which has significantly improved outcomes in both the (neo)adjuvant [4,5,6,7,8] and the metastatic setting [9,10]. The more recent gene-expression assays allow clinicians to assess the risk of recurrence in early breast cancer (EBC) [11,12,13], as well as to predict potential benefit from adjuvant chemotherapy [14,15,16,17]. In many patients found to have a disease with favorable gene-expression profile, chemotherapy could be avoided; however, a significant population of EBC patients may still be overtreated. Precision oncology aims at identifying the optimal treatment for each patient, specifically tailored to each unique cancer profile and to each individual health status in order to maximize survival and quality of life. Omics sciences are instrumental for this aim (Figure 1).

## 2. Metabolomics and NMR

Metabolomics, one of the latest -Omic sciences, entails the comprehensive characterization of the ensemble of endogenous and exogenous metabolites presents in a biological specimen. Metabolites simultaneously represent the downstream output of the genome, the transcriptome, and the proteome, as well as the upstream input from various external factors such as environment, lifestyle, diet, and drug exposure [18]. As a consequence, in the last few years, metabolomic phenotyping has been extensively applied in biomedical research.

Nuclear Magnetic Resonance spectroscopy (NMR) and mass spectrometry are the two most widely used analytical platforms for metabolomics. These two techniques can be considered complementary, since the weaknesses of one platform are compensated by the strengths of the other [19]. In contrast to the approach typically adopted in mass spectrometry, which is focused on target metabolites of interest, NMR metabolomics is usually performed using a high-throughput, untargeted approach, which provides a complete picture of all metabolites present or quantifiable in the sample above the NMR detection limit (concentrations > 1 μM) [19,20]. To date, NMR metabolomics are increasingly used for successful patient stratification in various diseases, and it provided unique insights into the fundamental causes of several physiological and pathophysiological conditions [21,22,23,24,25,26,27,28,29,30,31,32,33,34,35].

In principle, any biospecimen (i.e., cells, biofluids, and tissues) can be analyzed via NMR. The most common biological fluids analyzed by metabolomics are blood serum/plasma, urine, and saliva, as they can be collected with low invasiveness, and yield plentiful in biological information. Blood derivatives contain all the molecules that are secreted by different tissues in response to different physiological stimuli, conditions, or stressors [36]. Due to its important systemic role, the concentrations of metabolites in the blood are strongly controlled by feedback cycles, so serum/plasma samples are not subjected to extreme daily variations and can give information at a systemic level. Conversely, urine essentially contains metabolic waste, and thus is more affected by diet, environment lifestyle, and drug administration, resulting in significant day-to-day variability [37]. Saliva is an important physiological fluid that contains a highly complex mixture of substances, and it reflects both the systemic status [38] and the local health condition of the oral cavity [39]. A number of other local biofluids, such as exhaled breath condensate [40,41,42], cerebrospinal fluid [43,44], amniotic fluid [45], bile [46], synovial fluid [47,48], seminal fluid [49], and fecal extracts [50] can also be analyzed to investigate the metabolome of specific compartments. Cell lysates, cell growth media, and extracts of tissues can also be analyzed [51,52]. Further, the development of high resolution (HR) ^1^H magic angle spinning (MAS) spectra [53] has made viable the acquisition of data on small slices of tissue without the need of any extraction or pre-treatment [54,55,56].

Metabolomic fingerprints, as well as the identification and quantification of the most abundant metabolites (metabolomic profiling), can be directly obtained by the analysis of basic one-dimensional (1D) NMR spectra. ^1^D NOESY [57], ^1^H CPMG (Carr–Purcell–Meiboom–Gill) [58], and ^1^H diffusion-edited [59] are the pulse sequences most commonly used in metabolomics studies. NOESY spectra enables the detection of all molecules present in the sample above the NMR detection limit, CPMG spectra allow the selective detection of low molecular weight metabolites, whereas diffusion-edited spectra permit the observation of only high-molecular weight macromolecules (i.e., proteins and lipoproteins). The latter two sequences are particularly useful in biofluids such as serum/plasma that contain high amounts of both low and high molecular weight compounds.

Limiting the analysis to the most common biofluids employed in metabolomics, the number of molecules detectable and quantifiable by 1D-NMR span from slightly more than ten in breath condensate, to more than one hundred in urine. Assignment is mostly based on literature data, public databases, such as the de-facto reference standard Human Metabolome Database (HMDB) [60,61,62], commercially available databases and profiling software (i.e., ChenomX, AssureNMR). Spectra acquired at high magnetic field and two-dimensional experiments can be non-routinely employed in selected samples to identify unknown metabolites or to confirm NMR assignment [63]. Remarkably, besides small metabolites, serum and plasma also contain lipoproteins that with appropriate software (i.e., the Bruker IVDr platform) can be finely analyzed to derive, from serum and plasma NMR spectra, about 100 different lipid parameters that describe the distribution and analytical composition of lipid main fractions and subclasses [64]. This is especially important in the lipidomics domain, because the composition of lipoproteins has a strong influence on disease development, including BC [65].

If applied for population screening, NMR-based metabolomics/lipidomics could become a powerful clinical tool in precision oncology. However, to permit experimental reproducibility among different studies and/or different collection centers, it is extremely important that metabolomic data are collected under rigorously controlled standard operating procedures (SOPs). SOPs need to be strictly followed in all the main steps involved in the metabolomic work-flow, including sample collection, preanalytical processing, and storage [66,67,68]; NMR spectra recording [69]; and data/metadata compilation, description and storage [70].

**Figure 2:**
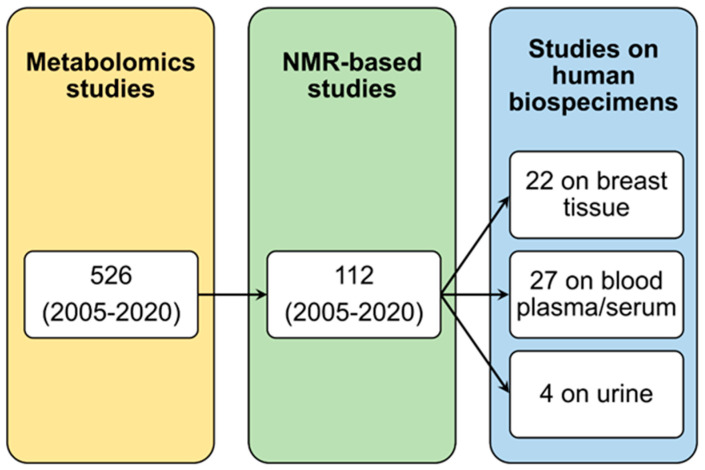
Selection of the scientific articles included in this NMR-based metabolomics review. The figure shows the workflow of the papers’ selection.

In this review, we will present an overview on the current status of NMR-based metabolomics studies in the setting of breast cancer using three different biological samples: breast tissue, serum/plasma and urine (Figure 2, Table 1). The translation in the clinical practice and the future perspectives for this analytical approach will be also examined and discussed.

The scientific publications reviewed in the present article were identified by database searching in three electronic databases [National Library of Medicine (Medline via PubMed^®^), Web of Science and Scopus] without any restriction on date of publication or publication status. Keywords were used as follows: (“metabolomics” OR “metabonomics) AND (“NMR” OR “nuclear magnetic resonance spectroscopy”) AND (“breast cancer”) AND (“biospecimen”, where biospecimen is tissue or plasma or serum or urine). The results of the searches were manually refined in order to remove non pertinent articles. In addition, previous systematic reviews were checked to ensure complete data collection.

## 3. NMR Metabolomics of Breast Tissue

High resolution magic angle spinning (HR-MAS) NMR spectroscopy allows the quantification of approximately 40 metabolites with a safe, non-destructive method that requires minimal sample preparation. Since HR-MAS analyzes intact tissue, it offers the potential to further characterize the same specimen via histopathology or utilizing transcriptomics and/or proteomics [71,90]. Several studies (Table 2) have shown that HR-MAS is able to discriminate between malignant and normal breast tissue [72,73], and between in-situ and infiltrating carcinoma [74]. Two studies have shown that the metabolic profile does not differ significantly based on intra-tumoral location and biospecimen type [75,76].

### 3.1. Correlation with Clinicopathological Factors

Metabolomics has been shown to be capable of predicting the status of BC prognostic factors such as estrogen receptor (ER), progesterone receptor (PR), and axillary lymph nodes (Table 3) [77]. In the study of Choi et al. [78], higher choline levels were found to correlate with ER-negative and PR-negative tumors. In addition, triple negative status (i.e., the absence of ER, PR, and HER2 receptors) was associated with higher choline-to-creatine and total choline-to-creatine ratios. In a study of Cao et al. [79], the metabolomic characterization of triple negative tumors confirmed higher choline levels, but also showed an association with lower creatine and glutamine levels, together with higher levels of glutamate, glycine, and lactate (Table 3). Tayyari et al. [80] performed a metabolic analysis to identify the potential differences between triple negative and hormone receptor-positive tumors, within both African-American and Caucasian patients. African-American patients with triple negative tumors showed higher concentrations of choline, glutamine, and glutathione compared to patients with hormone receptor-positive tumors. Conversely, Caucasian patients with triple negative tumors showed lower levels of glutamine in comparison with African-American patients with triple negative tumors.

In the context of HER2-positive tumors, Choi et al. [78] showed a significant correlation with higher levels of taurine, scyllo-inositol, and myo-inositol. Moreover, Cao et al. [79] described an association with higher concentrations of creatine, succinate, glycine and glutamine, and lower concentrations of alanine.

Choline-containing compounds have been found to be correlated with tumor grade and the proliferative marker Ki67. Choi et al. [78] showed that phosphocholine-to-creatine ratio was significantly greater in high grade and highly proliferative tumors. In addition, Ki67 was associated with increased phosphatidylcholine (PC) and total choline levels. In a different study published in 1998, a higher lactate-to-choline ratio was significantly correlated with high grade tumors [81]. Axillary lymph node involvement was associated with increased glycine and phosphocholine, and reduced betaine and taurine in a study by Bathen et al. [82].

Sitter et al. [83] showed that higher choline and glycine concentrations are characteristic of tumor larger than 2 cm as compared with smaller tumors. A later analysis of the same group [84] correlated the metabolic profile of 29 intact BC samples with clinical prognosis. Patients with an estimated good prognosis, defined by the absence of disease in axillary lymph nodes, primary tumors smaller than 2 cm, and ER- and PR-positive disease, were found to have a trend toward a lower concentration of glycine compared to those patients with poor prognosis. Moreover, the metabolomic analysis of tissue samples with a high proliferation index correlated with low concentrations of glucose.

### 3.2. Correlation with Response to Neoadjuvant Therapy

Metabolic profiling of breast tumor tissue using HR-MAS has been correlated with pathological response to neoadjuvant therapy in several studies (Table 2). In the study by Choi et al. [85], patients who achieved a pathological complete response (pCR) following neoadjuvant chemotherapy and subsequent surgery were compared with patients without a pCR result. No significant differences in the metabolite concentration of pre-treatment samples were found between responders and non-responders. Moreover, the metabolomic profile was not able to predict pCR prior to neoadjuvant treatment in a study of Euceda et al. [86]. However, pre-treatment biopsies of responders showed lower levels of glucose and higher levels of lactate compared with non-responders. Responders also showed an increase in glucose, lactate, and glutamine levels after treatment, and a decrease in phosphocholine, choline, and succinate. Cancer cells preferentially switch from anaerobic to aerobic glycolysis as result of the Warburg effect [122]. This phenomenon is associated with rapid glucose consumption and increased lactate production. As such, the lower levels of glucose and higher lactate found in pre-treatment samples of responders could reflect a more malignant metabolic profile that paradoxically also makes cells more sensitive to chemotherapy. The increase of glucose observed after treatment may be an expression of lower glucose consumption.

Cao et al. [87] showed that the pre-treatment levels of total choline (tCho) were higher in patients with tumors responsive to neoadjuvant chemotherapy than those with non-responsive tumors. Moreover, there was a reduction of tCho levels from pre-treatment to post-treatment samples in patients with partial response while this was not observed in patients with stable disease. However, these differences were not statistically significant. Conversely, glycerophosphorylcholine (GPC) was significantly decreased in post-treatment samples of patients in the responder group. The tCho signal is involved in cellular membrane turnover, therefore a decrease in tCho levels after treatment could suggest lower cellular proliferation.

### 3.3. Correlation with Survival

In the study by Giskeodegard et al. [88], the metabolic profile of BC tissue was correlated with 5-year survival rates. Higher levels of lactate and glycine were found to be associated with worse prognosis in patients with ER-positive BC undergoing upfront surgery without any prior treatment. This was not observed in the ER-negative subgroup, likely due to the small number of patients (*n* = 24), whilst also reflecting the metabolic differences between ER-positive and ER-negative tumors.

Similar results were found by Cao et al. [89]. In this study, increased levels of lactate on post-treatment tumor samples were associated with worse prognosis (survival < 5 years), while reduced levels of glycine and choline containing compounds correlated with better prognosis. Patients disease-free after five years of follow up also showed increased levels of glucose in response to treatment, in comparison with non-survivors. Conversely, pre-treatment metabolic analysis of tumor samples gave no prognostic information, suggesting that the difference observed between survivors and non-survivors resulted from a metabolic response to treatment. In this study, the impact of ER status on metabolic profile variations in response to treatment was not investigated.

Debik et al. [92] analyzed tissue samples from 132 women undergoing neoadjuvant chemotherapy. The metabolic profile of tumor biopsies detected during treatment was predictive of 5-year survival. In concordance with previous studies, patients with short survival had higher lactate and glycine levels in comparison with disease-free patients at five years. Increased lactate levels after treatment may reflect the activation of aerobic glycolysis and tumor response to hypoxia that led to high tumor aggressiveness and poor prognosis. Conversely, decreased glycine and tCho levels in response to treatment may be related to altered glycolysis and reduced cell proliferation, as an expression of lower disease aggressiveness and better prognosis.

### 3.4. Correlation with Transcriptomics and Proteomics

Metabolomics has been combined with transcriptomics and proteomics to better characterize breast tumors and to identify the mechanisms underlying BC heterogeneity.

Metabolite, gene expression, and protein data from 228 BC samples were analyzed by Haukaas et al. [90]. At the time of sample collection, patients had not received any treatment. HR-MAS identified three distinct metabolic clusters (MC1, MC2, and MC3). MC1 was characterized by the highest levels of GPC and phosphocholine (pCho); glucose was the most concentrated metabolite in MC2; glycine, alanine and lactate were predominant in MC3. These three clusters showed different expression of genes involved in glycolysis, gluconeogenesis, and glycerophospholipid metabolism, and genes related to extracellular matrix. They also expressed different cancer-related proteins. However, there were no significant differences in the distribution of PAM50-characterized molecular subtypes between the clusters. In a previous study merging transcriptomics and metabolomics [71], three subgroups of luminal A tumors with different metabolic profile and gene expression were identified. Thus, this supports the premise that metabolomics adds relevant information to transcriptomics and proteomics, in turn contributing to a more refined subclassification of breast tumors.

### 3.5. Correlation with Quantitative Conventional Breast Imaging

In the study by Yoon et al. [91], 53 BC specimens derived from pre-treatment core needle biopsies (CNB) were analyzed with HR-MAS. The metabolomic profile of each lesion was then correlated with conventional quantitative breast imaging parameters. It was shown that patients with high signal enhancement ratio (SER) at MRI with dynamic contrast enhanced (DCE), and with high FDG uptake value (SUV) at PET-CT scan, had higher levels of phosphatidylcholine (PC), choline and glycine. Choline was significantly correlated with SER, while PC correlated with SUV. Both these correlations were justified by the role of choline and PC in cell membrane synthesis, required for tumor cell replication and angiogenesis. High SER and SUV levels have been related to poor prognostic markers; therefore, choline and PC could be promising metabolites to be used to predict poor prognosis.

## 4. NMR Metabolomics of Blood Plasma/Serum

Circulating blood metabolites and lipoproteins may not only reflect the tumor metabolism, but more likely may provide a systemic picture of the fine balance between the tumor and the host metabolism considering the global physiological and immunological conditions of each patient with BC. For all these reasons, several aspects of the NMR-based metabolomic signature of BC in plasma/serum have been explored as providing novel insight into the molecular aspects of this disease.

### 4.1. Characterization of the Metabolomics Profile of BC Patients

Blood NMR-based metabolomics have been shown to have potential of distinguishing patients with BC with respect to healthy controls (HC) with high discrimination accuracies [94,95,98,111,115]. The levels of several circulating amino acids, and glyco- and lipo-proteins, have been shown to be statistically significantly altered in patients with BC (Table 4), implying a disruption of energetic homeostasis and amino acid metabolism to support cancer growth and evolution [94,95]. Recently, Jobard et al. [100] reported perturbations in circulating plasma metabolites prior to a breast cancer diagnosis in a population of 791 breast cancer cases and 791 matched controls. These alterations involved particularly histidine, N-acetyl glycoproteins (NAC), glycerol, and ethanol, but are statistically significant only in the premenopausal subgroup.

The metabolome of specific BC molecular subtypes has been also investigated. Study of the metabolomic profile of patients with triple-negative BC has further refined the molecular characterization of this BC subtype that accounts for 10–22% of all diagnosed BC and has the worst survival rate [115]. A recent study on plasma unravels how ER status impacts on the metabolomic profiles of patients with HER2-positive BC, with metabolomic data also studied in association with levels of circulatory cytokines [99]. Blood metabolomics has also shown how high expression of the receptor for the inositol 1, 4, 5 Trisphosphate, of which deregulation promotes tumor growth and aggressiveness, influences the host system metabolome by increasing lipoprotein content and the levels of lactate, lysine, and alanine and by decreasing the levels of pyruvate and glucose [111].

Important efforts have been made in order to describe the differences on plasma/serum metabolome across EBC and metastatic breast cancer (MBC) [97,105,108,110,113]. These two groups of patients can be discriminated by NMR metabolomics with high accuracy, and as reported in Table 4, several metabolites showed statistically different levels in patients with EBC and MBC, implying a progressive disruption and rewiring of several metabolic pathways following the evolution of the disease.

### 4.2. Blood Metabolomics: Prognosis and Risk of Relapse

Of interest to clinicians is the potential of metabolomics from a prognostic point of view. Metabolomics could provide the ability to discern between patients with EBC at high risk of recurrence, and those who may be cured by locoregional therapy alone. In the current era of precision medicine, this would represent an invaluable tool for clinicians, who may in turn offer more aggressive adjuvant therapies to the former group and sparing the latter from treatments whose benefit–risk ratio is poor [123,124]. In 2010, the first evidence supporting the usefulness of metabolomics as a potential biomarker of recurrence was published by Asiago and coauthors [40]. In this retrospective analysis, a PLS-DA model built using 11 metabolites provided a sensitivity of 86% and a specificity of 84% in discriminating patients with previous EBC free from disease at six years and patients with disease relapse. Of note, 55% of patients were correctly predicted to develop recurrence about 13 months before the clinical diagnosis of the same.

Over the past years our group has pursued this research line establishing a reproducible method of quantifying individual serum metabolomic fingerprints and demonstrating, in monocentric and multicentric cohorts of patients, its ability to accurately discriminate between MBC and EBC [105,109,110,113]. Furthermore, our data have shown that patients with EBC classified as “metastatic” on the basis of their metabolomic fingerprints presented high risk of disease recurrence. Thus, we hypothesized that EBC patients with occult micro-metastatic disease may already have features of the metastatic signature in their metabolomic fingerprint, and that this signature may be predictive for relapse. Following this approach, in a monocentric cohort of ER negative EBC patients we were able to predict cancer relapse with 82% accuracy [113]. These results have been reproduced obtaining 71% predictive accuracy by analyzing serum samples collected in several centers in South-East Asia, as a part of an unrelated Phase III adjuvant trial, from an heterogenous group of patients with mainly ER positive EBC [105]. Moreover, we have demonstrated that the serum NMR-based metabolomic fingerprinting approach can be effectively utilized to further refining the genomic risk of relapse predicted using the OncotypeDX 21-gene expression assay risk recurrence score [109].

### 4.3. Pharmacometabolomics in Breast Cancer Setting

The application of metabolomics for the study of drug effects and response—the so-called pharmacometabolomics—can contribute to personalized drug therapy [125], with relevant examples of its applications in the setting of BC already having been published. The primary aim of metabolomics in this context is to predict which patients will benefit most from a specific treatment. First in 2012, our group demonstrated that metabolomics may play a role in identifying patients with MBC with HER2-positive disease with a greater sensitivity to paclitaxel plus the anti-HER2 agent lapatinib [112]. Jiang and colleagues utilized NMR-based pharmacometabolomics to predict response to gemcitabine/carboplatin chemotherapy in a population of 29 patients with MBC. Baseline serum levels of formate and acetate were identified as potential predictive biomarkers of chemotherapy response [106]. In postmenopausal BC women treated with chemotherapy, the combination of lactate, alanine, and glucose has been associated with cancer progression [104]; moreover, high basal lactate levels were correlated with weight gain in postmenopausal women receiving chemotherapy [101].

More recently, some metabolomics studies have focused their attention on neoadjuvant chemotherapy (NAC). In breast cancer, NAC has become the approach of choice for patients with large primary tumors and for locally advanced disease [126]. The neoadjuvant approach offers the advantage of downstaging disease and reducing the size of tumors prior to surgery, thus making patients with inoperable tumors candidates for surgical resection or enabling breast-conserving surgery rather than mastectomy [92,126]. However, less than 30% of patients overall show complete pCR to NAC [114], with lower rates of response found in ER-positive, HER2-negative disease. Published metabolomic studies have been targeted at predicting response to NAC to enable the development of personalized treatment protocols, and at characterizing the effects of NAC on the metabolome [92,99,107,114]. Plasma/serum metabolomics has been shown to be effective in predicting pCR in different NAC regimes [99,114]. Moreover, it has been demonstrated that NAC induces relevant changes in patient metabolism during treatment, and that these alterations also persist some weeks after the completion of systemic therapy [92,107]. In particular, in the study conducted by Jobard et al. [107], the effects of trastuzumab and everolimus in combination were associated with alterations that involve several metabolic pathways reflecting a systemic effect, particularly on the liver and visceral fat.

### 4.4. NMR Lipidomics in Breast Cancer

Lipidomics represents a relatively new and promising complement to the more classical NMR metabolomics. In this particular setting, MS has been for a long time the preferred technology, however recent advancements on NMR analysis of blood plasma and serum have permitted its wider use. The Bruker IVDr Lipoprotein Subclass Analysis platform™ (Bruker Biospin) has enabled a fast and reliable quantification of the main lipoprotein parameters and their subfractions. This tool utilizes a chemometric approach based on a PLS regression model to perform lipoprotein subclass analysis on ^1^H NMR NOESY spectra [64,127].

The lipoproteins analysis via NMR was capable of providing further insights into the host metabolic alterations induced by different clinicopathological factors: HDL subfraction contents were strongly associated with PgR expression, whereas Ki67 expression was inversely associated with HDL phospholipids. Conversely no correlation was observed between lipoproteins and ER expression. This metabolic information could be relevant to characterize breast tumor aggressiveness and prognosis [116]. Moreover, it has been observed that women characterized by lower plasma levels of lipoproteins, lipids, glycoproteins, acetone, glycerol-derived compounds, and unsaturated lipids present a higher risk of developing BC over time [95].

Relevant alterations of the lipoproteins’ profiles of BC patients were also observed in association with chemotherapy treatments. In particular, alterations of HDL, LDL, VLDL cholesterols and triglycerides were observed during and after treatments. These observations were hypothesized to be related to inflammation processes and lipids homeostasis [107,117].

## 5. NMR Metabolomics of Urine

Although urine samples can be easily and non-invasively collected in large volumes, and require minimal pre-analytical and analytical preparation, the NMR-based urinary metabolome of patients with BC is relatively unexplored to date. Indeed, database research located only four published research articles.

In 2010, Slupsky and coauthors [121] described for the first time the urinary metabolic phenotype of a population of 48 patients with BC via NMR. Patients with BC in comparison to controls showed significantly lower levels of several metabolites (Table 5). However, the BC group was very heterogenous in terms of histologic types (including both invasive ductal and lobular carcinoma, as well as ductal carcinoma in situ), lymph node status (10 patients had at least one positive lymph node), and age (ranging from 30 to 86). These factors, if not properly considered, can present significant confounding factors. To date, other three research articles (Table 1) have been published [118,119,120] comparing the metabolic profiles of patients with BC to those of healthy controls. These studies confirmed the reduction of excretion levels of several metabolites, with the exception of citrate which showed a controversial trend (Table 5). The study by Men and coauthors [119] also examined the urinary levels of heavy metals, with As, Cd, and Cr significantly increased in the urine of patients with BC compared to controls. This finding suggests that urine concentrations of heavy metals and BC development could be associated.

Although these published results are thought provoking and point to relevant metabolite dysregulations in patients with BC, no large-scale study—mono- or multicenter—has been performed to date. Moreover, clinically relevant markers and outcomes (i.e., cancer stage, cancer recurrence, response to therapy) have never been explored via urine metabolomics.

## 6. Translation of NMR-Based Metabolomics in Clinics

This review aimed at highlighting the relevant results obtained using metabolomics by NMR in the BC setting and the possible role of this approach in the clinical practice.

BC is the most common type of cancer and the second most common cause of death in women worldwide [128]. Early detection and prompt treatment has been associated with a significantly improved prognosis observed over time in patients with BC.

The serum tumor markers, CEA and CA 15.3, are routinely used in therapy monitoring and follow up of patients with BC; conversely, their sensitivity and specificity for early diagnosis are poor [129]. Mammography is considered the gold standard in BC screening, however it has a sensitivity of 86.9% with relevant variability depending on tissue density and age [130].

Malignant tumors are characterized by increased gluconeogenesis, glycolysis, and fat mobilization, and decreased protein synthesis. The results described in the previous paragraphs show that these metabolic changes peculiar to malignant neoplastic change can be detected by metabolomics. Metabolomics is able to discriminate between cancer and normal breast tissue from the same patient with accuracy, sensitivity, and specificity around 90% [73]. Moreover, the metabolite analysis of blood and urine samples from BC patients differs significantly from healthy controls [94,95,98,111,115,131,132]. This evidence offers potential for the use of metabolomics, a minimally invasive technique, for early diagnosis of BC in the general population [133].

BC is a heterogeneous disease with high variability in prognosis and response to treatment driven by genetic, epigenetic, and phenotypic differences. The identification of the mechanisms underpinning this heterogeneity support the development of new drugs targeted to specific subgroup of patients, with the final aim to improve patient outcome. Transcriptomics and proteomics have attempted to classify breast tumors according to gene expression (intrinsic molecular subtypes—[134]) and protein expression (RPPA subtypes—[135]). As shown in the previous sections of this review, metabolomics can provide additional information to these -omics, leading to a deeper tumor characterization. ER and HER2 status are well estimated by metabolite analysis [79]. In addition, metabolomics can identify metabolic clusters within breast tumors, not reflecting the intrinsic molecular subtypes, but presenting significant differences in gene expression and protein expression profiles, and unique susceptibility to metabolically targeted drugs [90].

Neoadjuvant chemotherapy is commonly used to treat BC, not only for downsizing tumors, but also for the potential to monitor individual drug response. Moreover, in selected molecular subtypes, the achievement of a pCR after neoadjuvant treatment correlates with excellent long-term outcomes and a lower risk of disease recurrence [136]. Currently HER2 positivity, triple negative subtype, high Ki67, and the presence of tumor infiltrating lymphocytes (TILs) are the biomarkers most frequently used in recommending neoadjuvant chemotherapy. Predicting response to chemotherapy can spare patients with unresponsive disease from unnecessary side effects. Metabolomics was shown to play a role in predicting response to NAC.

We have summarized in this review that metabolomic profiling of serum samples collected before neoadjuvant chemotherapy was able to predict response in two small cohorts of patients. The first cohort was unselected for molecular subtype [114], while the second included only HER2-positive breast tumors [137]. The potential role of metabolomics in predicting response to treatment was also evaluated on breast tumor tissue. This analysis demonstrated that tumor metabolism changed significantly in response to neoadjuvant treatment. Metabolomic analysis on post-treatment tissue samples was able to discriminate between patients who experienced disease response to treatment and those who had non-responsive cancer. However, metabolomic analysis of pre-treatment tumor biopsies was not predictive probability of response to chemotherapy [85,86,87].

Developing prognostic biomarkers is one of the focuses of metabolomics in BC. Clinicopathological features are used to predict the risk of recurrence or development of metastatic disease. More recently, gene-expression assays such as Oncotype DX and Mammaprint have been introduced in clinical practice to refine risk estimation and prediction from adjuvant chemotherapy. However, these assays are time consuming, expensive, and can overestimate the risk of recurrence [138]. In addition, they are estimated on the primary tumor tissue and cannot identify the presence or absence of occult micro-metastases. Metabolomics can contribute to overcoming these limitations. As already detailed in the above paragraphs, our group developed a metabolomic score that classified patients as high or low risk of recurrent disease on the basis of the degree of metabolomic similarities with MBC fingerprints [105,113]. A high metabolomic score correlates with increased risk of recurrence and worse disease-free survival. Moreover, this metabolomic risk score can be used to sub-stratify the three Oncotype DX risk categories [109].

However, how far are we now from adopting NMR-based metabolomics as a population-wide screening method? The conceptual distance from the present situation to this ambitious goal is still wide, but it can be bridged by working in two directions: first it is necessary to standardize both the pre-analytical and the analytical procedures. Indeed, the biochemical composition of biospecimens is affected by how samples are collected, stored, prepared, and analyzed, and consequently differences in these steps can be particularly detrimental in multi-center studies [139]. Specifications for pre-examination processes for metabolomics in urine, venous blood serum and plasma have been already published by CEN (CEN/TS 16945:2016) [140]; however, these recommendations are still not universally employed. Secondly, to increase the robustness and the reliability of the results already provided, well-planned, large-scale, multicenter, population-based studies in which all heterogeneous BC patient groups are well represented are needed. NMR-based metabolomics is a fast, high-throughput, robust, and reproducible technique, thus moving from the analysis of hundreds to thousands of samples is realistically an approachable target [19,141].

## 7. Conclusions

The NMR-based metabolomics studies presented in this review have demonstrated that a metabolic signature of BC exists and can be detected in breast tissue, blood serum/plasma, and urine. This approach has the potential to improve early diagnosis of BC, to allow early prediction of recurrence and estimating prognosis, and to further stratify the heterogenous spectra of BC patients and the individual response to (neo)adjuvant treatments. Metabolomics by NMR can play a pivotal role in precision oncology and it is mature enough to support, and eventually sub-stratify, the identification of risk groups obtained by clinical and genomic tools already in use.

## Figures and Tables

**Table 1:** List of evaluated publications.

Ref.	Biospecimen	Population Study (*n*)	Cohort Allocation	EBC/MBC	ER Status	HER2 Status	Mean Age (Yrs)	NMR (MHz)
Borgan et al., 2010 [71]	T	46 BC	Trondheim (Norway)	46 EBC	41 ER+/5 ER−	Not reported	64	600
Li et al., 2011 [72]	T	31 (13 BC; 18 HC)	Seoul (South Korea)	13 EBC (11 IC; 2 DCIS)	11 ER+/2 ER−	12 HER2+/1 HER2	50	500
Bathen et al., 2013 [73]	T	228 BC	Trondheim (Norway)	228 EBC	168 ER+/49 ER−	Not reported	60.7	600
Chae et al., 2016 [74]	T	60 BC	Seoul (South Korea)	60 EBC (30 DCIS; 30 DCIS + IC)	40 ER+/20 ER−	4 HER2+/36 HER2−	48.7	400
Park et al., 2016 [75]	T	31 BC	Seoul (South Korea)	31 EBC (IC)	21 ER+/10 ER−	23 HER2+/8 HER2−	54.2	600
Gogiashvili et al., 2018 [76]	T	18 BC	Oberhavel (Germany)	18 EBC	Not reported	Not reported	Not reported	600
Giskeødegård et al., 2010 [77]	T	160 BC	Trondheim (Norway)	160 EBC (IC)	119 ER+/39 ER−	Not reported	62	600
Choi et al., 2012 [78]	T	34 BC	Seoul (South Korea)	34 EBC (IC)	26 ER+/6 ER−	5 HER2+/27 HER2−	52.2	500
Cao et al., 2014 [79]	T	75 BC	Trondheim (Norway)	75 EBC (IC)	44 ER+/31 ER−	30 HER2+/45 HER2−	64	600
Tayyari et al., 2018 [80]	T	82 (47 BC; 35 HC)	Multicenters USA	47 EBC (44 IC; 3 DCIS)	29 ER+/18 ER−	47 HER2+/0 HER2−	Not reported	800
Cheng et al., 1998 [81]	T	19 BC	Boston (USA)	19 EBC (18 IC;1 DCIS)	Not reported	Not reported	60	400
Bathen et al., 2007 [82]	T	77 BC	Trondheim (Norway)	77 EBC (IC)	62 ER+/15 ER−	Not reported	62	600
Sitter et al., 2006 [83]	T	85 (83 BC, 1 LC, 1 HC)	Trondheim (Norway)	83 EBC	Not reported	Not reported	62	600
Sitter et al., 2010 [84]	T	29 BC	Trondheim (Norway)	29 EBC (IC)	18 ER+/11 ER−	Not reported	Not reported	600
Choi et al., 2013 [85]	T	37 BC	Seoul (South Korea)		25 ER+/12 ER−	14 HER2+/25 HER2−	50.5	500
Euceda et al., 2017 [86]	T	122 BC	Trondheim (Norway)	122 EBC (IC)	101 ER+/21 ER−	122 HER2−	49	600
Cao et al., 2012 [87]	T	30 BC	Trondheim (Norway)	30 EBC (IC)	27 ER+/3 ER−	Not reported	62	600
Giskeødegård et al., 2012 [88]	T	98 BC	Trondheim (Norway)	98 EBC (IC)	71 ER+/24 ER−	Not reported	69	600
Cao et al., 2012 [89]	T	85 BC	Trondheim (Norway)	80 EBC, 5 MBC (IC)	50 ER+/34 ER−	Not reported	49	600
Haukaas et al., 2016 [90]	T	228 BC	Oslo (Norway)	228 EBC (224 IC; 4 DCIS)	178 ER+/40 ER−	26 HER2+/192 HER2−	55.5	600
Yoon et al., 2016 [91]	T	53 BC	Seoul (South Korea)	53 EBC (IC)	36 ER+/17 ER−	12 HER2+/41 HER2−	49.6	600
Debik et al., 2019 [92]	T, S	118 BC	Oslo (Norway)	118 EBC (IC)	100 ER+/18 ER−	118 HER2−	48.9	600
Bro et al., 2015 [93]	P	838 (419 BC; 419 HC)	Denmark	not reported	not reported	not reported	not reported	600
Cala et al., 2018 [94]	P	58 (29 BC; 29 HC)	Bogotà (Colombia)	29 EBC (19 IDC; 10 ILC)	19 ER+/10 ER−	6 HER2+/23 HER2−	51	400
Lecuyer et al., 2018 [95]	P	602 (206 BC; 396 HC)	France	not reported	not reported	not reported	49.3	500
Louis et al., 2015 [96]	P	145 (73 BC; 72 HC)	Hasselt (Belgium)	73 EBC (61 IDC; 11 ILC; 1 DCIS)	62 ER+/11 ER−	not reported	58.5	400
Richard et al., 2017 [97]	P	65 BC	Mons (Belgium)	50 EBC (IC); 15 MBC	not reported	not reported	57.6	500
Suman et al., 2018 [98]	P	122 (72 BC; 50 HC)	Lucknow (India)	not reported	not reported	not reported	44.3	800
Vignoli et al., 2020 [99]	P	43 BC	Aviano (Italy)	43 EBC (IC)	22 ER+/21 ER−	43 HER2+	49	600
Jobard et al., 2021 [100]	P	1582 (791 BC; 791 HC)	Lyon (France)	791 EBC (685 IC; 69 DCIS)	EBC: 536 ER+/100 ER−	Not reported	56.8	600
Keun et al. [101]	S	21 BC	London (England)	Not reported	Not reported	Not reported	59	600
Asiago et al., [102] 2010	S	56 BC	Houston (TX, USA)	56 EBC (IC)	26 ER+/25 ER−	not reported	53.7	500
Gu et al., 2011 [103]	S	57 (27 BC; 30 HC)	Detroit (MI, USA)	not reported	not reported	not reported	55.9	500
Stebbing et al., 2012 [104]	S	88 BC	London (England)	13 EBC; 75 MBC	64 ER+/24 ER−	34 HER2+/54 HER2−	59	600
Hart et al., 2017 [105]	S	699 BC	International	590 EBC (IC); 109 MBC	EBC: 552 ER+/37 ER−	EBC: 108 HER2+/388 HER2−	41.5	600
Jiang et al., 2018 [106]	S	29 BC	Singapore	29 MBC	not reported	6 HER2+/7 HER2−	52.7	800
Jobard et al., 2017 [107]	S	79 BC	France	79 BC	not reported	79 HER2+	50.5	800
Jobard et al., 2014 [108]	S	190 BC	Lyon (France)	104 EBC; 86 MBC	not reported	32 HER2+/156 HER2−	57.1	800
McCartney et al., 2019 [109]	S	115 BC	New York (USA)	28 MBC; 87 EBC (IC)	115 ER+	115 HER2−	54	600
Oakman et al., 2011 [110]	S	140 BC	Prato (Italy)	89 EBC (IC); 51 MBC	111 ER+/29 ER−	28 HER2+/108 HER2−	57	600
Singh et al., 2017 [111]	S	42 (27 BC; 15 HC)	Lucknow (India)	27 EBC (IC)	not reported	not reported	58.6	800
Tenori et al., 2012 [112]	S	579 BC	International	579 MBC	not reported	not reported	not reported	600
Tenori et al., 2015 [113]	S	175 BC	New York (USA)	95 MBC; 80 EBC (IC)	62 ER+/110 ER−	47 HER2+/126 HER2−	53	600
Wei et al., 2013 [114]	S	28 BC	Tübingen (Germany)	28 EBC	19 ER+/9 ER−	13 HER2+/15 HER2−	47.9	600
Wojtowicz et al., 2020 [115]	S	95 (9 BC; 86 HC)	Wroclaw (Poland)	not reported	9 ER−	9 HER2−	56.67	600
Flote et al., 2016 [116]	S	56 BC	Norway	56 EBC (IC)	52 ER+/4 ER−	3 HER2+/53 HER2−	55.1	600
Madssen et al., 2018 [117]	S	60 BC	Norway	56 EBC (4 DCIS; 56 IC)	52 ER+/4 ER−	3 HER2+/53 HER2−	55.4	600
Zhou et al., 2017 [118]	S; U	22 (11 BC; 11 HC)	Xi’an (China)	10 EBC (IC); 1 MBC	not reported	not reported	58	600
Men et al., 2020 [119]	U	144 (106 BC; 38 HC)	Tengzhou (China)	106 EBC (IC)	not reported	not reported	50.6	600
Silva et al., 2019 [120]	U	78 (40 BC; 38 HC)	Funchal (Portugal)	not reported	not reported	not reported	59	400
Slupsky et al., 2010 [121]	U	170 (48 BC; 50 OC; 72 HC)	Edmonton (Canada)	37 IDC; 7 DCIS; 4 ILC	not reported	not reported	56	600

P: Plasma; S: Serum; U: Urine; T: tissue; BC: breast cancer; HC: healthy controls; IDC: invasive ductal carcinoma; ILC: Invasive lobular carcinoma; DCIS: ductal carcinoma in situ; EBC: early breast cancer; MBC: metastatic breast cancer; LABC: locally advanced breast cancer; HER2+: human epidermal growth factor receptor 2 positive; RBC: relapsed breast cancer; NRBC: non-relapsed breast cancer; TNBC: Triple-negative breast cancer.

**Table 2:** List of altered metabolite levels identified in breast tissue of breast cancer patients to study their metabolomic profiles.

Metabolite	BC vs. CTR					IC vs. DCIS	Poor Prognosis vs. Good Prognosis					GR vs. PR					Changes in Response to Treatment	High SER/SUV vs. Low SER/SUV
												Pre-Treatment			Post-Treatment			
	[72]	[73]	[80]	[81]	[83]	[74]	[78]	[84]	[88]	[89]	[92]	[85]	[86]	[87]	[86]	[87]	[89]	[91]
Choline			↑									↓	↑		↓			↑
Phosphatidylcholine/creatine												↓						
Total choline	↑	↑								↑				↑		↓	↓	
Phosphatidylcholine			↑	↑	↑				↑			↓	↑		↓		↓	↑
Glycine		↑					↑	↑	↑	↑	↑	↓	↑		↓		↓	↑
Scyllo-inositol							↑											
Myo-inositol						↓												
Glycerophosphocholine					↓					↓		↓	↑		↓	↓	↓	
Creatine		↑							↓				↑		↓			
Glutamine															↑			
Glutamate													↑					
Taurine	↑	↑							↓	↓		↓	↑		↓			
Alanine						↓							↑		↓			
Ascorbate		↑											↑					
Lactate									↑	↑	↑		↑		↑			
Succinate						↓							↑		↓			
Methionine			↑															
Uridine			↑															
Lipids			↓															
Unsatured lipids			↓															
ATP			↓															
Glycerophosphocholine/hosphatidylcholine					↓													
Glycerophosphocholine/choline					↓													
Phosphatidylcholine/choline					↑													
Glucose		↓							↓				↓		↑		↑	
Glutathione													↑					
Glycerophosphocholine/choline						↓												

BC: breast cancer; CTR: control; IC: invasive carcinoma; DCIS: ductal carcinoma in situ; PR: poor responders; GR: good responders; SER: signal enhancement ratio; SUV: maximum standardized FDG uptake value. ↑/↓ higher/lower level in the first group of each comparison.

**Table 3:** List of altered metabolite levels identified in breast tissue of breast cancer patients to study clinicopathological factors.

Metabolite	ER+ vs. ER−				PR+ vs. PR−		HER2+ vs. HER2−		High G vs. Low G			TN vs. NonTN			N+ vs. N0		T > 2 cm vs. T < 2 cm	High Ki67 vs. Low Ki67	
	[77]	[78]	[79]	[82]	[77]	[78]	[78]	[79]	[78]	[53]	[83]	[78]	[79]	[80]	[82]	[83]	[83]	[78]	[84]
Choline	↓	↓	↓		↓	↓						↑	↑			↓	↑		
Choline/creatine												↑							
Total choline/creatine												↑							
Phosphatidylcholine/creatine									↑									↑	
Total choline																		↑	
Phosphatidylcholine	↑			↓	↓			↓					↑		↑	↑		↑	
Glycine	↓		↓	↓	↓			↑					↑		↑	↑	↑		
Scyllo-inositol				↓			↑												
Myo-inositol							↑									↑			
Glycerophosphocholine	↓			↑	↓						↑		↑			↓			
Creatine	↑				↓	↓		↑					↓			↓			
Glutamine			↑					↑					↓						
Glutamate			↓										↑						
Taurine	↑			↑		↓	↑								↓	↓			
Alanine	↓				↓			↓											
Ascorbate	↑				↓														
Lactate	↓		↓	↓	↓											↓			
Succinate								↑											
ATP														↓					
Lactate/Choline										↑									
Betaine																↓			
Glucose																↑			↓

↑/↓ higher/lower level in the first group of each comparison.

**Table 4:** List of altered metabolite levels identified in plasma/serum samples of BC patients to study several aspects of this pathology.

Metabolite	BC vs. CTR						ER+ vs. ER−	MBC vs. EBC						REL vs. NR		Response to Chemotherapy							
																PR vs. GR					Changes during Treatment		
	[115]	[98]	[95]	[94]	[111]	[100]	[99]	[97]	[98]	[105]	[108]	[110]	[113]	[105]	[102]	[112]	[106]	[92] (NAC)	[114] (NAC)	[99] (NAC)	[92] (NAC)	[92] (NAC + Bevacizumab)	[107] (Trastuzumab+ Everolimus)
3-hydroxy-2-Methyl-butanoic acid															↓								
3-Hydroxybutyrate										↑	↑				↓							↑	
Acetate										↑							↓				↓↑↑		↓
Acetoacetate	↑										↑										↓↑↓	↑	↓
Acetone	↓		↓																				↑
Alanine	↓			↑	↑					↑	↓												↓
Albumin Lysyl																							↓
Apo-B							↑													↑			
Arginine		↑	↑																				
Betaine											↓												↓
Cholesterol							↑													↑			
Choline										↑				↑	↓								↓
Citrate	↑																	↑			↓↓↓		↓
Creatine			↑							↑											↓↑↑		↓
Creatinine			↑				↑			↑											↓↑↑		↓
Dimethylglutarate																					↑↓↑		
Ethanol						↑														↑			
Formate		↓								↑					↓		↓				↓↓↓	↓	
Glucose	↑	↑	↑		↓			↑		↓		↑	↑			↓							↓
Glutamate	↓	↑			↑				↑	↑	↑			↑	↓	↑							
Glutamine	↑	↓	↑					↓		↓									↑				
Glycerol						↑					↑												
Glycerol-derived compounds			↓																				↑
Glycerophosphocholine																							↓
Glycine		↓							↓	↑				↑							↑↑↓		
Glycoproteins			↓																				
Histidine						↑				↑	↓		↓	↑	↓			↓	↓		↓↑↑		↓
Isoleucine		↓								↑				↑					↓	↑	↑↓↓		↓
Lactate	↓	↑		↑	↑			↓	↑	↑			↑	↑	↑						↑↓↓		
Leucine										↑				↑							↑↓↑	↑	
Linolenic acid																			↑				
Lipids		↑	↓	↑	↑						↑	↓	↑										↑
Lipoproteins		↑	↓																				↑
Lysine		↑	↑		↑			↓				↑									↑↓↑		↓
Mannose											↑												↑
Methanol																							↓
Methionine										↑											↓↑↑		
Myo-Inositol																							↓
N-acetyl glycoproteins		↑			↓	↑			↑		↑												↑
N-Acetyl-Cysteine												↑											
N-Acetyl-Glycine															↓								
Nonanedioic acid															↓								
Ornitine																					↓↑↑		
Phenylalanine										↑	↑	↑		↑		↑		↓			↓↑↑		↓
Phospholipids							↑													↑			
Proline										↑		↑			↓								↓
Pyruvate					↓			↓			↑										↓↓↑		
Threonine																			↑				
Triglycerides																				↑			
Tyrosine	↓	↑								↑			↑	↑	↑								↓
Unsaturated lipids			↓																				
Valine		↓	↑	↑										↑						↑	↓↑↑		↓

BC: breast cancer; CTR: control; ER: estrogen receptor; PR: poor responders; GR: good responders; NAC: neoadjuvant chemotherapy; MBC: metastatic BC; EBC: early BC; REL: relapse; NR: no relapse. ↑/↓ higher/lower level in the first group of each comparison.

**Table 5:** List of altered metabolite levels identified in urine samples of BC patients with respect to healthy controls.

Metabolite	Studies on Urine Samples			
	[119]	[120]	[121]	[118]
2-oxoisocaproate	↓			
3-methylglutarate	↓			
4-cresol sulphate		↓		
4-hydroxyphenylacetate			↓	
acetate		↓	↓	
acetone		↓		
alanine	↓	↓	↓	
asparagine			↓	
betaine		↓		
carnitine		↓		
choline		↓		
cis-aconitate		↓		
citrate		↓		↑
creatine		↓	↓	
creatinine	↓	↓	↓	
dimethylamine	↓	↓	↓	
ethanolamine			↓	
formate		↑	↓	
glucose			↓	
glutamate (n-acetylaminoacides)	↓			
glutamine	↓	↓		
glycine	↓	↓		
guanidoacetate		↓		↓
hippurate	↓		↓	
histamine	↓			
hypoxanthine		↓		
isoleucine	↓		↓	
lactate		↓	↓	
leucine	↓		↓	
levoglucosan			↓	
lysine	↓			
malonate	↓			
mannitol		↓		
methylhistidine	↓			
phenylacetylglycine				↓
pyroglutamate			↓	
pyruvate		↓		
serine		↓		
succinate		↓	↓	
sucrose			↓	
taurine	↓	↓		
threonine		↓	↓	
trans-aconitate			↓	
trigonelline		↓		
trimethylamine n-oxide	↓	↓		
uracil			↓	
urea			↓	
valine	↓	↓	↓	
α-hydroxybutyrate		↑		
α-hydroxyisobutyrate		↓		
α-oxoglutarate		↓		
β-hydroxyisobutyrate	↓			
β-hydroxyisovalerate		↓		

↑/↓ higher/lower level in the first group of each comparison.
